# High Levels of Oxidative Stress Create a Microenvironment That Significantly Decreases the Diversity of the Microbiota in Diabetic Chronic Wounds and Promotes Biofilm Formation

**DOI:** 10.3389/fcimb.2020.00259

**Published:** 2020-06-03

**Authors:** Jane H. Kim, Paul R. Ruegger, Elyson Gavin Lebig, Samantha VanSchalkwyk, Daniel R. Jeske, Ansel Hsiao, James Borneman, Manuela Martins-Green

**Affiliations:** ^1^Molecular, Cell and Systems Biology Department, University of California, Riverside, Riverside, CA, United States; ^2^Microbiology and Plant Pathology Department, University of California, Riverside, Riverside, CA, United States; ^3^Statistics Department, University of California, Riverside, Riverside, CA, United States

**Keywords:** wound healing, impaired healing, wound microbiome, dysbiosis, *Pseudomonas aeruginosa*, *Enterobacter cloacae*, probiotics, skin microbiome

## Abstract

Diabetics chronic wounds are characterized by high levels of oxidative stress (OS) and are often colonized by biofilm-forming bacteria that severely compromise healing and can result in amputation. However, little is known about the role of skin microbiota in wound healing and chronic wound development. *We hypothesized* that high OS levels lead to chronic wound development by promoting the colonization of biofilm-forming bacteria over commensal/beneficial bacteria. To test this hypothesis, we used our *db/db*^−/−^ mouse model for chronic wounds where pathogenic biofilms develop naturally after induction of high OS immediately after wounding. We sequenced the bacterial rRNA internal transcribed spacer (ITS) gene of the wound microbiota from wound initiation to fully developed chronic wounds. Indicator species analysis, which considers a species' fidelity and specificity, was used to determine which bacterial species were strongly associated with healing wounds or chronic wounds. We found that healing wounds were colonized by a diverse and dynamic bacterial microbiome that never developed biofilms even though biofilm-forming bacteria were present. Several clinically relevant species that are present in human chronic wounds, such as *Cutibacterium acnes, Achromobacter* sp., *Delftia* sp., and *Escherichia coli*, were highly associated with healing wounds. These bacteria may serve as bioindicators of healing and may actively participate in the processes of wound healing and preventing pathogenic bacteria from colonizing the wound. In contrast, chronic wounds, which had high levels of OS, had low bacterial diversity and were colonized by several clinically relevant, biofilm-forming bacteria such as *Pseudomonas aeruginosa, Enterobacter cloacae, Corynebacterium frankenforstense*, and *Acinetobacter* sp. We observed unique population trends: for example, *P. aeruginosa* associated with aggressive biofilm development, whereas *Staphylococcus xylosus* was only present early after injury. These findings show that high levels of OS in the wound significantly altered the bacterial wound microbiome, decreasing diversity and promoting the colonization of bacteria from the skin microbiota to form biofilm. *In conclusion*, bacteria associated with non-chronic or chronic wounds could function as bioindicators of healing or non-healing (chronicity), respectively. Moreover, a better understanding of bacterial interactions between pathogenic and beneficial bacteria within an evolving chronic wound microbiota may lead to *better solutions for chronic wound management*.

## Introduction

The human skin is an important organ that functions as an interface between the human body and the environment. The skin is inhabited by a complex and diverse community of microbes whose impact on health and disease is still under investigation (Grice et al., 2009; Grice and Segre, 2011; Hannigan and Grice, 2013). The skin microbiota consists of both benign commensal bacterial species and opportunistic pathogens that are able to cause infection and disease if not controlled (Grice and Segre, 2011). Commensal bacteria have recently been shown to stimulate the adaptive and innate immune system and directly or indirectly prevent pathogens from causing infections (Grice and Segre, 2011; Byrd et al., 2018; Meisel et al., 2018; Coates et al., 2019).

Long-term bacterial infections are a characteristic of chronic wounds. These wounds develop when regulation of wound healing processes is defective or does not occur. Chronic wounds are characterized by high levels of oxidative stress (OS) and chronic inflammation that cause extensive damage to the host tissue. This is due to a continuous influx of inflammatory cells that release cytotoxic enzymes, increasing free oxygen radicals and resulting in cell death (Chodorowska and Roguś-Skorupska, 2004; Schäfer and Werner, 2008; Stewart and Franklin, 2008; MacLeod and Mansbridge, 2016). The healing process for chronic wounds is complicated by pathogenic bacteria which take advantage of host nutrients that are leeched in the destructive inflammatory microenvironment and contribute to the damaging of the host tissue when they form biofilm (Gjødsbøl et al., 2006; James et al., 2008; Schäfer and Werner, 2008; Zhao et al., 2013; Misic et al., 2014; Wolcott et al., 2016; Loesche et al., 2017). These biofilms are recalcitrant to conventional antibiotic therapies because the structure of the biofilm decreases the efficacy of antibiotic therapy by significantly decreasing their diffusion rate. The biofilm extracellular polymeric substance (EPS) also helps the bacteria in the biofilm evade the host innate and adaptive immune system (Gontcharova, 2010; Dhall et al., 2014; Raghav et al., 2018).

Bacterial biofilms are composed of a matrix of EPS, extracellular DNA, proteins/peptides, and lipids surrounding bacterial cells that are attached to abiotic and biotic surfaces (Fux et al., 2005; Dowd et al., 2008b; James et al., 2008; Zhao et al., 2013; Nouvong et al., 2016). *In vivo*, biofilms are composed of a multitude of aerobic and anaerobic bacterial species across the phylogenetic tree that aggregate into sessile microcolonies (Burmølle et al., 2010). Bacteria within the biofilm differ significantly from their planktonic counterparts in their morphology, mode of communication, and metabolism (Davey and O'toole, 2000; Omar et al., 2017). The biofilm provides a unique environment to facilitate bacterial cell-to-cell signaling by the production of and detection of quorum-sensing molecules, which promote collective behavior such as optimizing nutrient acquisition and regulation of virulence, leading to sustained pathogenicity in the wound (Stewart and Franklin, 2008; Zhao et al., 2013). At the same time, stochastic processes and non-uniform gene expression can lead to the appearance of subpopulations of bacteria with different phenotypes and environmental responses (Stewart and Franklin, 2008).

Interactions between different bacterial species are very complex in both biofilm and free-living states. In the presence of biofilm-forming bacteria, commensal bacteria can be out-competed for resources and eradicated (Giaouris et al., 2015). Similarly, benign colonizers of normal skin microbiota can participate in multi-species biofilm production and sustain a high burden of infection (Hotterbeekx et al., 2017). Biofilms in chronic wounds are complex, capable of harboring many species of bacteria, each with very different nutritional demands and roles (Leaper et al., 2015; Phalak et al., 2016). Common wound-associated bacteria such as *Staphylococcus, Streptococcus*, and *Pseudomonas* can produce exotoxins that cause broad damage to host tissue, destruction of host cells, and disruption of normal cellular metabolism leading to further tissue necrosis (Hotterbeekx et al., 2017). An increased burden of infection can increase the risk of amputation needed to stop the spread the infections in patients with chronic wound (Frykberg and Banks, 2015; Olsson et al., 2019).

Chronic wounds in humans can develop when any process of the wound healing program, which includes homeostasis, inflammation, proliferation, and remodeling, is disrupted or impaired. Patients with one or more underlying pathological conditions, such as metabolic diseases, are particularly at risk for developing chronic wounds (Olsson et al., 2019). For example, injuries in Type II diabetes patients can develop into chronic wounds; in the foot, these are called diabetic foot ulcers (DFUs) (Reiber et al., 1998). With over ~30 million Americans with diabetes, DFUs are a major health concern and cost the US health care industry $13 billion a year, since ~25% will experience a foot ulcer during their lifetime (Loots et al., 1998; Stadelmann et al., 1998; Raghav et al., 2018). DFUs must be treated frequently and diligently because they are commonly infected with bacterial biofilms that prevent healing. Amputation of the lower extremities may be necessary if the wound is unable to heal and the infection and biofilm cannot be controlled. These patients have a 50% mortality risk within 5 years of amputation, attributed to the pathophysiology of diabetes and other co-morbidities such as obesity (Gurney et al., 2018).

To investigate how bacteria participate in chronic wound development, we used our murine *db/db*^−/−^ diabetic chronic wound model (Dhall et al., 2014; Kim et al., 2019). This model is characterized by obesity, diabetes, chronic inflammation and lack of angiogenesis that result in impaired healing. To create the chronic wounds, we treated the mice once, immediately after wounding, with inhibitors of the antioxidant enzymes catalase and glutathione peroxidase. The inhibition of these two enzymes results in high levels of OS. In the *db/db*^−/−^ chronic wounds, biofilm starts to form around 3 days after wounding and induction of OS in the wound tissue. The wounds became fully chronic within 20 days and contained mature biofilm, comprised of bacteria and extracellular polymeric substance (Dhall et al., 2014; Li et al., 2020), also found in human diabetic chronic wounds.

We have previously shown that OS levels correlate inversely with the levels of diversity of the microbiota in the wound using the Shannon diversity index. This index measures both the species richness (number of taxa) and evenness (relative abundances) of each of the species. Low indices indicate lower diversity, found typically in infections (e.g., one microorganism dominates and causes disease). High indices indicate higher diversity, found typically in stable, healthy communities. We found that the level of OS significantly contributed to a difference in Shannon diversity. The greatest difference in diversity was found between wounds with basal to low levels of OS and wounds with high levels of OS. The former showed high diversity and the latter low diversity (Kim et al., 2019).

Based on these findings, *we hypothesized* that high levels of OS create a microenvironment that significantly decreases the diversity of the microbiota in diabetic chronic wounds and stimulates biofilm-forming bacteria to grow and form biofilm. We show here that in non-chronic wounds, the microbiota of the wound is as diverse as that of the skin and the bacteria do not form biofilm even though biofilm-forming bacteria are present in the wound during healing. In contrast, chronic wounds have much less bacterial diversity in comparison with the microbiota of the skin, and are strongly colonized by biofilm-forming bacteria. These findings suggest that bacteria found in non-chronic wounds may benefit or assist in wound healing, and/or participate in the exclusion of pathogenic biofilm-producers.

## Materials and Methods

### Reagents

3-Amino-1,2,4-triazole (ATZ) from Tokyo Chemical Industry Co., Ltd. (Portland, OR). Mercaptosuccinic acid (MSA) from Sigma-Aldrich (St. Louis, MO). Buprenorphine from Henry Schein (Dublin, OH). Isoflurane from Henry Schein (Dublin, OH). Tegaderm Film 1624W from 3M (Maplewood, MN).

### Chronic Wound Model

All experiments were completed in accordance and compliance with federal regulations and University of California policy and all procedures have been approved by the University of California, Riverside Institutional Animal Care and Use Committee (IACUC). The detailed description of how to obtain chronic wounds in *db/db*^−/−^ mice has been previously published by us (Dhall et al., 2014; Kim and Martins-Green, 2019). Briefly, *db/db*^−/−^ mice were bred in house from B6.BKS(D)-*Lepr*^*db*^/J heterozygotes catalog #000697 from the Jackson Laboratories. The mice were housed in a conventional non-barrier vivarium and after wounding they were housed in separate cages. Control and experimental groups were age-matched and both males and females were used in the study indiscriminately because both genders form chronic wounds similarly (Dinh and Veves, 2008; Navarro-Peternella et al., 2016). A total of 77 mice were used for this experiment. Only 5–6 month-old *db/db*^−/−^ that weighed at least 50 g were used. The hair was cut short with an electric shaver and then removed with a chemical depilatory 24 h prior to wounding. A wound was created on the back of *db/db*^−/−^mice by first gently wiping the skin with 70% ethanol and then excising one 7-mm full-thickness skin biopsy punch under isoflurane anesthesia. Buprenorphine, an analgesic, was administered by intraperitoneal injection at 0.05 mg buprenorphine/kg of mouse weight in sterile phosphate buffer solution (PBS), given 20 min before surgery and 6 h after the surgery. Tegaderm, which is a gas permeable bandage but cannot be penetrated by bacteria, was applied firmly to prevent contamination of the wound site. A non-chronic wound was created by treating the wound with the vehicle PBS. To create chronic wounds, OS in the wound tissue was increased by using specific inhibitors for catalase, ATZ, and glutathione peroxidase, MSA. ATZ was injected intraperitoneally at 1 g ATZ/kg of mouse weight in sterile PBS ~20 min before surgery. MSA was administered topically onto the wound between the Tegaderm and the wound site at 150 mg MSA/kg of mouse weight in sterile PBS 10 min after surgery.

### Bacterial Sampling and DNA Extraction

Bacterial samples were collected from wounds with a sterile cotton swab via the Levine method (Levine et al., 1976). Briefly, the Tegaderm was removed and a sterile cotton swab was rolled around 1 cm^2^ in the center of the wound for 10 s. A new piece of Tegaderm was placed securely on the wound and skin after sample collection. Bacterial swabs were collected at 0, 1, 2, 3, 5, 10, 15, and 20 days after wounding (D0, D1, D2, etc., respectively). The swabs were stored dry, without freezing media, in sterile microcentrifuge tubes at −80°C until DNA extraction. DNA extractions were performed on thawed swabs using the MOBio PowerSoil DNA Isolation Kit (which became the Qiagen PowerSoil DNA Isolation Kit) as described by the manufacturer, with a 90 s bead-beating step.

### Bacterial rRNA Internal Transcribed Spacer (ITS) Analysis

Illumina bacterial rRNA ITS gene libraries were constructed as follows: PCRs were performed in an MJ Research PTC-200 thermal cycler (Bio-Rad Inc., Hercules, CA) as 25 μl reactions containing, 50 mM Tris (pH 8.3), bovine serum albumin (BSA) at 500 μg/ml, 2.5 mM MgCl_2_, 250 μM of each deoxynucleotide triphosphate (dNTP), 400 nM of the forward PCR primer, 200 nM of each reverse PCR primer, 2.5 μl of DNA template and 0.625 units JumpStart Taq DNA polymerase (Sigma-Aldrich, St. Louis, MO). PCR primers targeted a portion of the small-subunit (ITS-1507F, GGTGAAGTCGTAACAAGGTA) and large-subunit (ITS-23SR, GGGTTBCCCCATTCRG) rRNA genes and the hypervariable ITS region (Ruegger et al., 2014), with the reverse primers including a 12-bp barcode and both primers including the sequences needed for Illumina cluster formation; primer binding sites were the reverse and complement of the commonly used small-subunit rRNA gene primer 1492R (Frank et al., 2008) and the large-subunit rRNA gene primer 129F (Hunt et al., 2006). PCR primers were only frozen and thawed once. Thermal cycling parameters were 94°C for 5 min; 35 cycles of 94°C for 20 s, 56°C for 20 s, and 72°C for 40 s; followed by 72°C for 10 min. PCR products were purified using a Qiagen QIAquick PCR Purification Kit (Qiagen, Valencia, CA) according to the manufacturer's instructions.

### Bacterial ITS Sequencing and Bioinformatics

DNA sequencing (single-end 150 base) was performed using an Illumina MiSeq (Illumina, Inc., San Diego, CA). Clusters were created using template concentrations 2.5 pM and PhiX at 107 K/mm^2^. Data processing was performed with USEARCH v10.0 (Edgar, 2010). The UPARSE pipeline for de-multiplexing, length trimming, quality filtering and operational taxonomic unit (OTU) picking using default parameters or recommended guidelines that were initially described (Edgar, 2013) and which have been updated at https://www.drive5.com/usearch/manual10/uparse_pipeline.html. Briefly, after demultiplexing and using the recommended 1.0 expected error threshold, sequences were trimmed to a uniform length of 149 bp and then dereplicated. Dereplicated sequences were subjected to error-correction (denoised) and chimera filtering to generate zero-radius operational taxonomic units (ZOTUs) using UNOISE3 (Edgar, 2016). An OTU table was then generated using the otutab command. ZOTUs having non-bacterial DNA were identified and enumerated by performing a local BLAST search (Altschul et al., 1990) of their seed sequences against the nucleotide database. ZOTUs were removed if any of their highest scoring BLAST hits contained taxonomic IDs within the Rodent family, Fungal or Viridiplantae kingdoms, or PhiX. Taxonomic assignments to bacterial ZOTUs were made by finding the lowest common taxonomic level of the highest BLAST hits excluding unclassified designations. Data were normalized within each sample by dividing the number of reads in each OTU by the total number of reads in that sample. The bacterial rRNA ITS sequences have been deposited in the National Center for Biotechnology Information (NCBI)'s Sequence Read Archive (SRA) under the BioProject Accession Number PRJNA623025.

### Statistics

Alpha diversity using the Shannon index was calculated with Qiime (version 1.9.1). One-way ANOVA in R (version 3.6.1) was used to calculate the significance of the bacterial alpha diversity in chronic and non-chronic wounds. Indicator species analysis (Dufrêne and Legendre, 1997) was used to classify bacterial species that were highly associated with either non-chronic wounds or chronic wounds. Calculations for the indicator values were done with indicspecies (version 1.7.6) (De Cáceres and Legendre, 2009). Statistical significance of the indicator values was tested with permutation tests using permute (version 0.9–5).

## Results

### Bacterial Colonization of Non-chronic and Chronic Wounds

**Figure 1:**
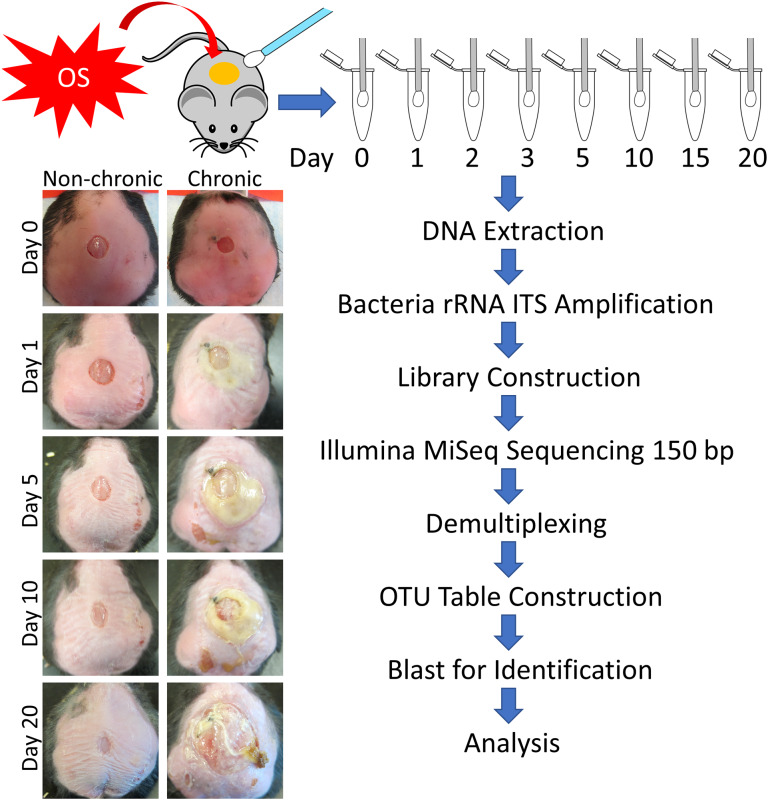
Flowchart of the experimental procedure to obtain bacterial counts and wound types. Sterile swabs were used to sample the microbiota from non-chronic and chronic wounds from injury until D20. Non-chronic wounds healed around day 20 whereas chronic wounds became fully chronic with biofilm formation by D20. After DNA extraction, the bacterial ITS rRNA region was PCR amplified for each sample. Libraries were sequenced by Illumina MiSeq with 150 bp single end reads, followed by demultiplexing and OTU table construction for bacterial identification.

We sampled the microbiome from 77 mice with either non-chronic or chronic wounds from wounding to 20 days post-wounding (Figure 1). The non-chronic wound cohort consisted of 40 mice and the chronic wound cohort consisted of 37 mice. The analysis of the wound microbiome was conducted by sequencing the ITS rRNA of the bacterial genome, a region of nucleotides that was flanked by the 16S and 23S rRNA genes. After 20 days, mice treated with placebo (PBS) undergo re-epithelization and heal. During the healing of non-chronic wounds, we observed a clear exudate, indicating the lack of biofilm formation and the analyses of the microbiota in the wound over time showed a diverse microbiome very similar to that of undamaged skin (data not shown). Conversely, wounds in mice treated with inhibitors for antioxidant enzymes (chronic wounds) showed yellowish fluid and thick biofilm on the wound and exhibited reduced microbiome diversity. Indeed, the microbiota was composed of a few bacteria species which are known to form strong biofilm.

**Figure 2:**
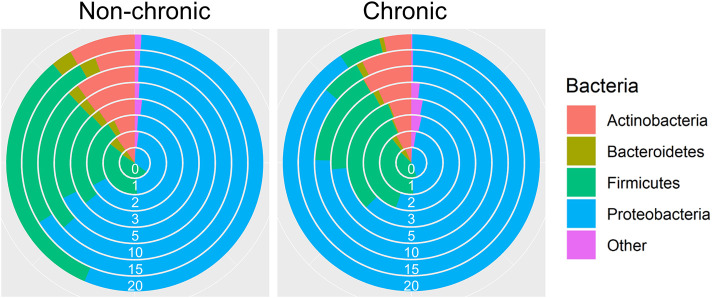
Bacterial diversity in the chronic wound model. Bacteria identified in the non-chronic and chronic wounds crossed phylum lines. Most of the bacteria sequenced were found in the Actinobacteria, Firmicutes and Proteobacteria phylum. While proportions of the phylum did not change much in non-chronic wounds as the wound healed, the proportion of Proteobacteria, which consisted of *P. aeruginosa* and *E. cloacae*, increased over time in chronic wounds. Non-chronic wound, *n* = 40; chronic wound, *n* = 37.

Over 50 million counts were categorized, representing ~8,000 OTUs with ~1,470 uniquely identified species. Three major phyla, *Actinobacteria, Firmicutes*, and *Proteobacteria*, comprised a large majority of the bacteria in non-chronic and chronic wounds (Figure 2). The average proportion of bacteria in each phylum remained relatively the same in non-chronic wounds; however, the proportion of the phyla changed in chronic wounds. The *Proteobacteria* population of bacteria in chronic wounds began to increase starting from D2 as the population of bacteria from *Actinobacteria* and *Firmicutes* decreased. Major contributors to the *Proteobacteria* populations were *Pseudomonas aeruginosa* and *Enterobacter cloacae*. They were found in ~23 non-chronic wounds and 18 chronic wounds and ~39 non-chronic wounds and 34 chronic wounds, respectively. *Achromobacter*, also belonging in the *Proteobacteria* group, was found in ~21 non-chronic and 10 chronic wounds. A *Firmicutes* species, *Staphylococcus xylosus*, was present in ~in 36 non-chronic wounds 25 chronic wounds.

**Figure 3:**
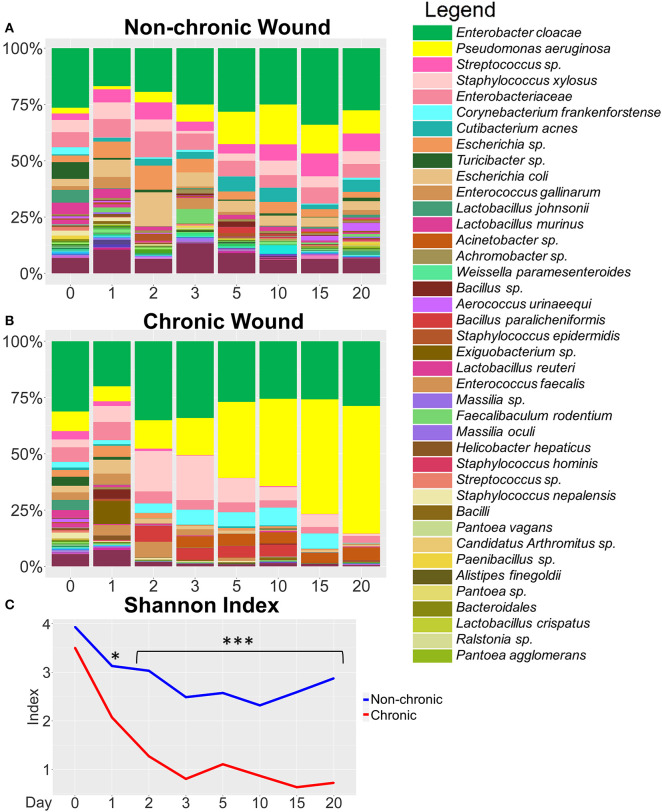
Bacterial composition in non-chronic and chronic wounds when *P. aeruginosa* was present in the microbiota. The average % relative abundance of bacterial OTUs was calculated across the top 100 OTUs for wound cohorts. The top 40 most abundant bacteria are shown in the legend. **(A)** Non-chronic wounds had a diverse bacteriome that included pathogenic bacterial species. However, biofilm formation was not visibly detected, and the wounds healed in ~20 days. **(B)** Chronic wounds were composed of a less diverse bacteriome and over time became dominated by a few pathogenic bacteria such as *P. aeruginosa* and *E. cloacae*. **(C)** Alpha diversity measured with Shannon Index confirmed that chronic wounds had much less bacterial diversity in the wounds compared to non-chronic wounds (p-value < 0.0001). Non-chronic wound, *n* = 20; chronic wound, *n* = 19. *p < 0.05, ***p < 0.001.

**Figure 4:**
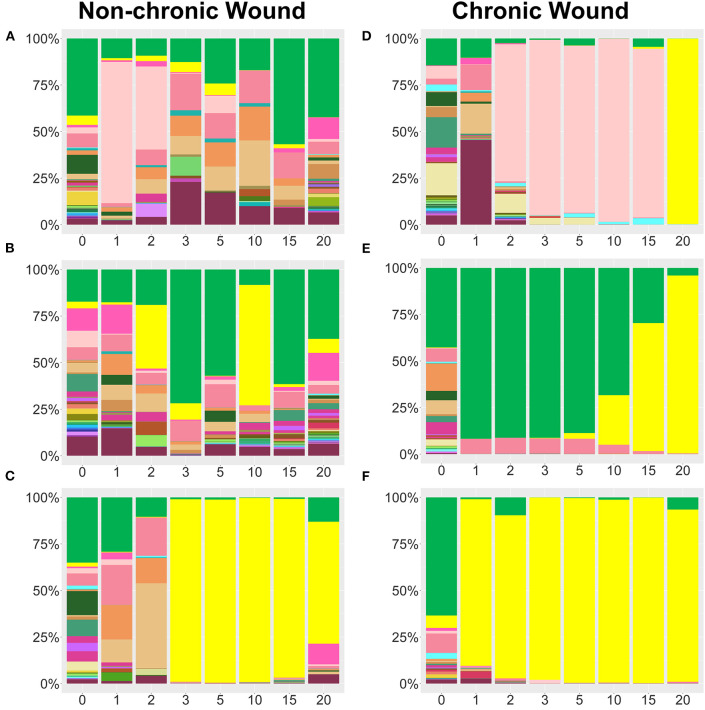
Examples bacterial profiles from non-chronic and chronic wounds. **(A–C)** Examples of non-chronic wound bacterial profiles three individual mice showed differences in bacterial percentages in the absence of high levels of OS in the wound. Species legend is shared with Figure 3. Non-chronic wounds may have pathogenic bacteria in the wound, but they don't form biofilm and the wounds will ultimately heal. *P. aeruginosa* was abundant in **(C)**, but, in the absence of high levels of OS, no biofilm formed. **(D–F)** Individual profiles of bacteria in chronic wounds show distinct bacteriome patterns as pathogenic bacteria colonize a wound in the presence of high levels of OS. *P. aeruginosa, S. xylosus*, and *E. cloacae* predominate over other bacteria and form biofilm as the chronic wounds develop.

**Figure 5:**
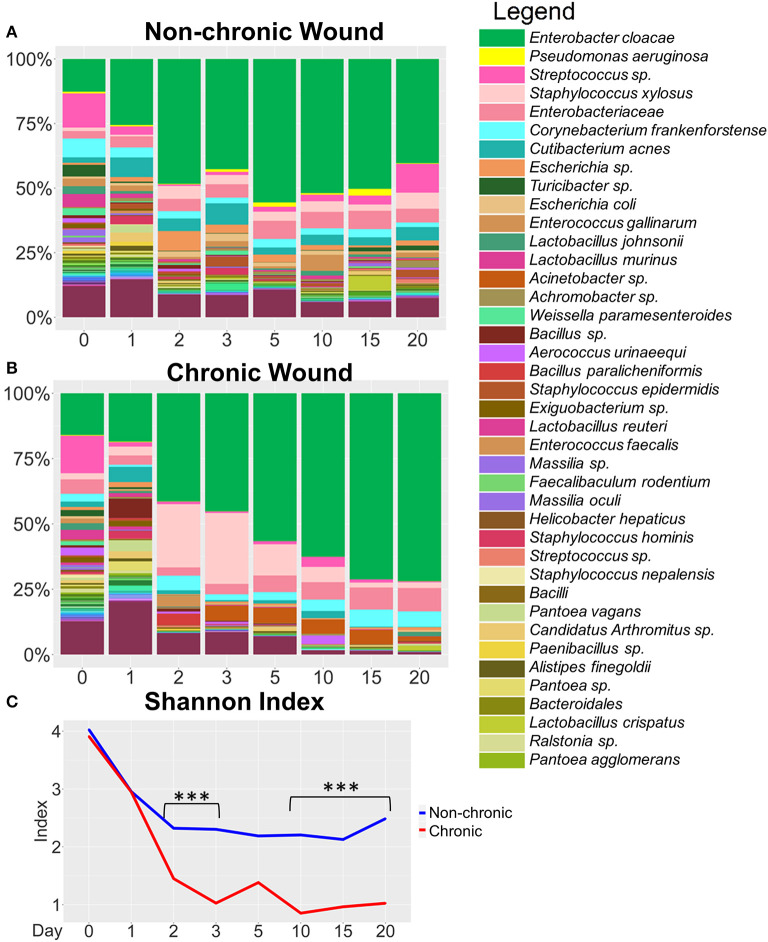
Bacterial composition in non-chronic and chronic wounds when *P. aeruginosa* was not present in the microbiota. **(A,B)** Average % bacterial composition was calculated for the top 100 OTUs for wound cohorts without *P. aeriugnosa* present in the skin microbiome. The top 40 most abundant bacteria are shown in the legend. **(A)** Non-chronic wounds have a diverse microbiome with strong colonization by *E. cloacae*, a biofilm forming bacteria yet the wounds do not develop biofilm. **(B)** Bacterial diversity in the chronic wounds decreases over time, with the wound becoming dominated by *E. cloacae*. **(C)** Alpha diversity measured by the Shannon Index shows that chronic wounds have less diversity in the wounds compared to non-chronic wounds (*p* < 0.0001). Diversity in non-chronic wounds decreases at start of wound healing and then stabilizes once the wound heals. Diversity in chronic wounds drops precipitously as bacterial infection leads to biofilm formation that harbors only a few species. Non-chronic wound, *n* = 20; chronic wound, *n* = 18. ***p < 0.001.

**Figure 6:**
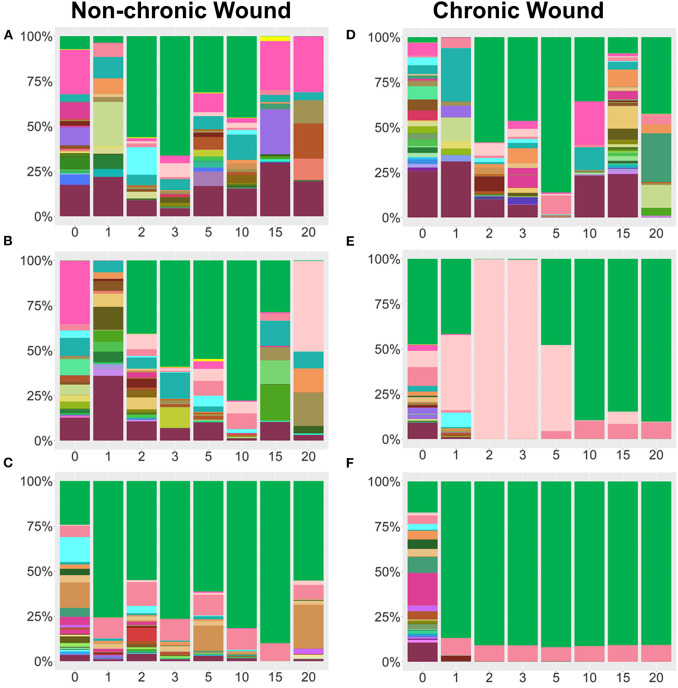
Examples of individual mice bacterial wound profiles from non-chronic and chronic wounds in the absence of *P. aeruginosa* in the bacteriome of the skin. **(A–C)** Examples of three individual non-chronic bacterial wound profiles show differences in percentages of the individual bacteria in the absence of high levels of OS in the wound. Species legend is shared with Figure 5. Despite of the abundance of *E. cloacae*, a biofilm forming bacterium, formation of biofilm does not occur, and wounds heal. **(D–F)** Individual profiles of bacteria in chronic wounds in the presence of high OS show distinct microbiome patterns with biofilm forming bacteria such as *E. cloacae* and *S. xylosus* predominating in the wound. These wounds contain biofilm. Non-chronic wound, *n* = 20; chronic wound, *n* = 18.

The collection of wound swabs was collected over ~2 years. About halfway through the sample collection, stricter biosecurity regulations were imposed on the conventional vivarium that housed the *db/db*^−/−^ mice. In normal vivarium conditions, the mouse skin microbiota contained high percentage of *P. aeruginosa* in the bacterial population whereas in the more stringent conditions the mice skin contained no more than 3% of *P. aeruginosa* in the bacterial population in the wound microbiota. In the former cohort, we analyzed the progression of the microbiota in 20 mice with non-chronic wounds and 19 mice with chronic wounds. In the latter cohort, we analyzed the skin microbiota of 20 mice with non-chronic wounds and 18 mice with chronic wounds for a total of 39 mice in normal condition of the vivarium and 38 mice in more stringent conditions. We describe our findings below in Figures 3–6.

### Bacterial Colonization of Chronic Wounds When *P. aeruginosa* Was Significantly Present in the Microbiota

At the species level, non-chronic wounds were populated with a diverse mixture of bacteria during wound healing (Figure 3A). Biofilm-forming bacteria, such as *P. aeruginosa, E. cloacae*, and *S. xylosus*, were detected starting from D0 (skin microbiota) through D20. In these non-chronic wounds, *P. aeruginosa* was present in the wound as the wound healed but never at more than 18% of the bacterial population. *E. cloacae* on the other hand represented more than 25% of the bacterial population in wounds at D0, D3, D5, D10, D15, and D20. *S. xylosus* constituted <10% of the population all through wound healing. Despite the presence of these three biofilm-forming bacteria in the non-chronic wounds, biofilm never formed in the wounds of these mice, and the wounds went on to heal and close in ~20 days. Other common bacteria found throughout the healing of non-chronic wounds were *Corynebacterium frankenforstense, Acinetobacter* sp. and *Staphylococcus epidermis* at very low relative abundance. These bacterial species have the potential to also form biofilm, but do not in healing non-chronic wounds. Many other bacterial species were found throughout the course of healing, including *Aerococcus urinaeequi, Enterococcus gallinarum, Cutibacterium acnes, Massilia oculi, Escherichia coli, Lactobacillus crispatus, Lactobacillus murinus*, and *Lactobacillus johnsonii*.

When wounds were treated with inhibitors for catalase and glutathione peroxidase to induce chronicity, a dynamic shift was observed in the wound-associated bacterial population (Figure 3B).Whereas, the population of *P. aeruginosa* was <20% of the wound associated community within the first 3 days after wounding, the population increased to >50% by D15 and continued to increase to D20. *E. cloacae* represented 20–30% of the wound-associated bacteria when *P. aeruginosa* was abundant.

We measured the alpha diversity of wound-associated communities using the Shannon diversity index (Figure 3C), a measure of both species' richness (number of taxa) and evenness, which is a measure of the relative abundances of each of the species. Low indices indicate lower levels of diversity, found typically in infections (e.g., one microorganism dominated and caused disease). High indices indicate higher levels of diversity, found typically in stable, healthy communities. This analysis showed that diversity in non-chronic wound microbiome was high in comparison to the chronic wound microbiome (*p* < 0.0001). After wounding, the diversity in non-chronic wounds decreased between D0 and D10. As the wound began to heal, the diversity of the microbiome began to increase again. Conversely, the bacterial diversity of chronic wounds with high levels of OS exhibited a precipitous decrease. By D3, once biofilm production was observed, the bacterial diversity in the wound continued to decrease and did not recover.

Whereas, relative abundance measurements showed general trends in bacterial population dynamics, striking inter-individual trends in bacterial communities were observed, despite the mice having the same genetic background and living in the same environment (Figure 4). Non-chronic wounds were colonized by many different species with the entire community exhibiting dynamic day-to-day variations (Figures 4A–C shows examples of wounds from three mice). The wounds of these mice healed well and in a timely manner. In one mouse, temporary blooms of *S. xylosus* and *E. cloacae* at D1, D2, D15, and D20 were observed (Figure 4A). In another mouse (Figure 4B), *P. aeruginosa*, whose population exceeded 25% of the bacterial population in D2 and even more in D10, was unable to dominate in the wound in the absence of high OS levels. No biofilm formation was observed (Figure 4B). In a third mouse, *P. aeruginosa* could colonize the non-chronic wound with ~98% relative abundance yet was unable to become biofilm-forming without high levels of OS (Figure 4C). Note that this profile was very similar in composition and trend of a chronic wound containing *P. aeruginosa* biofilm (Figure 4F).

Analysis of the bacterial composition in individual mice with chronic wounds showed evidence of bacterial interactions between *S. xylosus, E. cloacae*, and *P. aeruginosa* (Figures 4D–F). In these three examples, chronic wounds were strongly populated with *S. xylosus* and *E. cloacae;* however, these bacterial species were unable to compete for dominance when *P. aeruginosa* was present. In the first example, the chronic wound was dominated by *S. xylosus* starting from D2. Sometime between D15 and D20, there was a change in the dominating bacteria. Within 5 days, *P. aeruginosa* entirely out-competed *S. xylosus* from the wound bed (Figure 4D). In the second chronic wound, *E. cloacae* dominated the wound-associated community at D1. Starting from D5, *E. cloacae* was rapidly overtaken by *P. aeruginosa* (Figure 4E). In another chronic wound, the microbiome became populated by *P. aeruginosa* by D1. No other bacteria took over to form biofilm (Figure 4F). Numerous species identified in the chronic wound microbiome of our *db/db*^−/−^ mice are also represented in human chronic wounds (Table 1).

**Table 1:** Bacteria found in human chronic wounds.

**Bacteria**	**References**
*Acinetobacter* sp.	Gjødsbøl et al., 2006, 2012; Dowd et al., 2008a; James et al., 2008; Gontcharova, 2010; Wolcott et al., 2016
*Anaerococcus* sp.	Gardner et al., 2013; Smith et al., 2016; Wolcott et al., 2016
*Bacillus* sp.	Gjødsbøl et al., 2006; Dowd et al., 2008a,b; Gontcharova, 2010; Wolcott et al., 2016
*Corynebacterium* sp.	Gontcharova, 2010; Gjødsbøl et al., 2012; Gardner et al., 2013; Scales and Huffnagle, 2013; Smith et al., 2016; Wolcott et al., 2016
*Enterobacter* sp.	Gjødsbøl et al., 2006; Dowd et al., 2008a; James et al., 2008; Smith et al., 2016; Wolcott et al., 2016
*Enterobacter cloacae*	Gjødsbøl et al., 2006, 2012
*Enterococcus* sp.	Dowd et al., 2008a; James et al., 2008; Scales and Huffnagle, 2013; Smith et al., 2016; Wolcott et al., 2016
*Enterococcus faecalis*	Gjødsbøl et al., 2006, 2012; Wolcott et al., 2016
*Escherichia* sp.	Dowd et al., 2008a; James et al., 2008; Gontcharova, 2010; Gjødsbøl et al., 2012; Scales and Huffnagle, 2013
*Escherichia coli*	Gjødsbøl et al., 2006; Dowd et al., 2008a
*Finegoldia* sp.	Gontcharova, 2010; Gardner et al., 2013; Wolcott et al., 2016
*Finegoldia magna*	Smith et al., 2016; Wolcott et al., 2016
*Paenibacillus* sp.	Dowd et al., 2008a
*Peptoniphilus* sp.	Dowd et al., 2008a; Gardner et al., 2013; Smith et al., 2016; Wolcott et al., 2016
*Porphyromonas* sp.	Gardner et al., 2013
*Prevotella* sp.	Gontcharova, 2010; Gardner et al., 2013; Scales and Huffnagle, 2013; Smith et al., 2016; Wolcott et al., 2016
*Propionibacterium* sp.	Gontcharova, 2010; Wolcott et al., 2016
*Propionibacterium acnes*	Wolcott et al., 2016
*Pseudomonas* sp.	Gjødsbøl et al., 2006; Dowd et al., 2008a; James et al., 2008; Gontcharova, 2010; Scales and Huffnagle, 2013; Smith et al., 2016; Wolcott et al., 2016
*Pseudomonas aeruginosa*	Gjødsbøl et al., 2006, 2012; Scales and Huffnagle, 2013; Wolcott et al., 2016
*Staphylococcus* sp.	Dowd et al., 2008a; James et al., 2008; Gontcharova, 2010; Gardner et al., 2013; Smith et al., 2016; Wolcott et al., 2016
*Staphylococcus epidermidis*	Scales and Huffnagle, 2013; Wolcott et al., 2016
*Streptococcus* sp.	Dowd et al., 2008a; James et al., 2008; Gontcharova, 2010; Scales and Huffnagle, 2013; Smith et al., 2016; Wolcott et al., 2016
*Turicibacter* sp.	Wolcott et al., 2016

### Bacterial Colonization of Chronic Wounds When *P. aeruginosa* Was Present in the Microbiota at <3% of the Bacterial Population

A cohort of mice were identified to have *P. aeruginosa* present <3% in the wound microbiome (*n* = 38) (Figures 5, 6). In these mice, the strongest colonizer of the wound was *E. cloacae*, whose presence was found in high percentages in both non-chronic (*n* = 20) and chronic wounds (*n* = 18) (Figure 5). Except for D0, *E. cloacae* was present on average 25–55% in non-chronic wounds (Figure 5A). However, despite the species' strong presence in the non-chronic wounds, biofilm formation could not be visually identified, and the wounds healed in a timely manner. This indicated that *E. cloacae*, much like *P. aeruginosa*, was unable to form biofilm in a wound in the absence of high levels of OS. Other bacteria commonly found in non-chronic wounds when *P. aeruginosa* was virtually absent were *C. frankenforstense, Enterococcus faecalis, Streptococcus sp*., and *Bacillus paralicheniformis*. In chronic wounds, the colonization of the wound by *E. cloacae* was stronger with biofilm formation initiating between D3 and D5 (Figure 5B). In D0 and D1, the population of *E. cloacae* was 15-18%. However, starting from D2, the percent population began to increase. By D5, *E. cloacae* population had increased to 56%. After D10, the percent composition of *E. cloacae* in the wound began to stabilize with D15-20 wounds consisting of >70% abundance. Other bacterial species, such as *E. faecalis, B. paralicheniformis, L. murinus*, and *C. frankenforstense*, were found in the chronic wound with *E. cloacae*. However, their populations rarely exceeded 10%. Shannon index analysis between non-chronic wounds and chronic wounds showed that the diversity of the bacteria between the two types of wounds was significantly different (*p* < 0.0001) (Figure 5C).

Individual mouse profiles showed distinct bacterial trends in non-chronic and chronic wounds (Figure 6). In non-chronic wounds, *E. cloacae* could be found during healing and even when the wound is healed. Some wounds had *E. cloacae* in a few days after wounding (Figures 6A,B), while other wounds had *E. cloacae* colonization throughout healing (Figure 6C). However, biofilm formation was never observed in these wounds. In chronic wounds, the presence of *E. cloacae* was more common in higher % (Figures 6D–F). Most chronic wounds presented with strong colonization by *E. cloacae* (Figures 6E,F). Of those wounds, most showed complete domination of the wound with *E. cloacae* (Figure 6F) showing that without the presence of significant amounts of *P. aeruginosa, E. cloacae* had the ability to become a strong biofilm-forming colonizer. Specific bacterial interactions were also observed with *E. cloacae, S. xylosus*, and *Streptococcus* sp. (Figure 6E). In the absence of *P. aeruginosa, E. cloacae* was able to out-compete *S. xylosus* (Figure 6E) in a similar manner found with *P. aeruginosa* (Figure 4D).

### Bacteria That Colonize Chronic Wounds Can Be Used as Indicators of Chronicity

**Figure 7:**
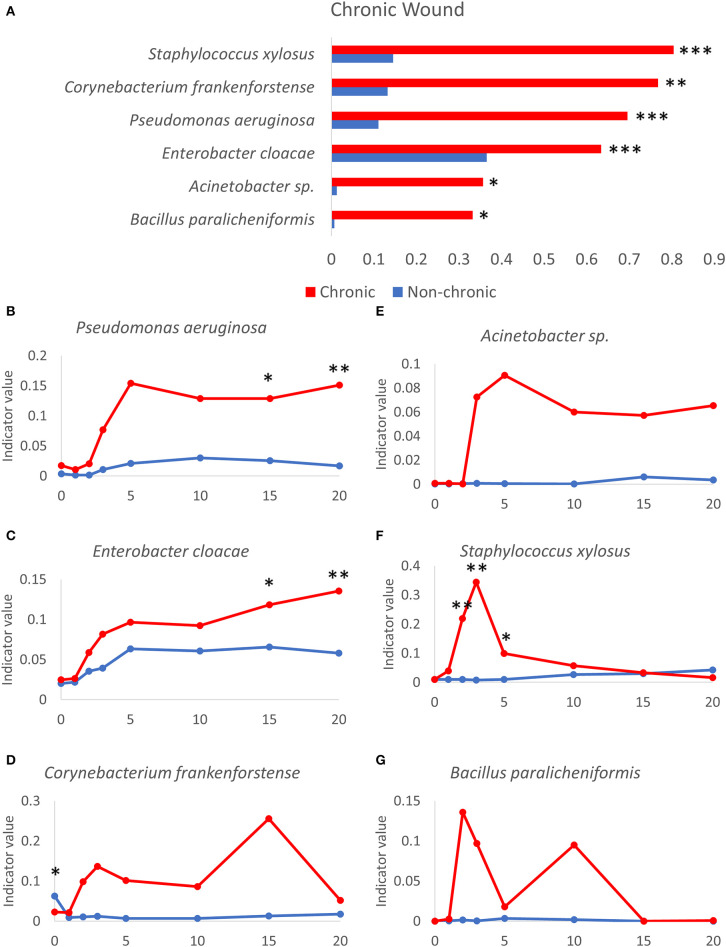
Bacteria that participate in the microbiome profile of chronic wound microenvironment. **(A)** Several bacteria that can form biofilm are highly indicative in chronic wounds compared to non-chronic wounds. Indicator values of non-chronic (blue lines) and chronic (red lines) wounds are shown for the following bacteria over time as chronic wounds develop. Gram-negative biofilm forming bacteria: **(B)** *P. aeruginosa*, **(C)** *E. cloacae*, **(D)** *C. frankenforstense*, **(E)** *Acinetobacter* sp., **(F)** *S. xylosus*. Gram-positive biofilm-forming bacteria: **(G)** *B. paralicheniformis*. Non-chronic wound, *n* = 40; chronic wound, *n* = 37. * *p* < 0.05, **p < 0.01, ***p < 0.001.

**Figure 8:**
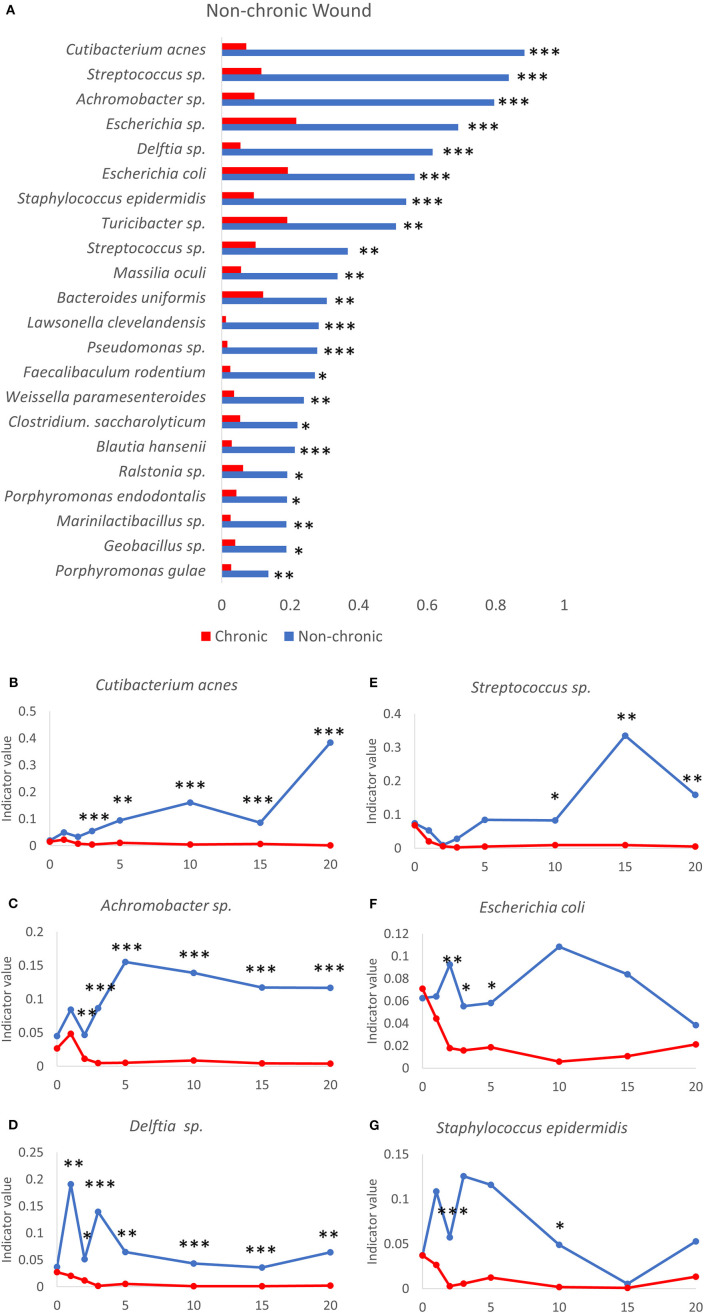
Bacteria that participate in a diverse microbiome profile typically found in the non-chronic wound microenvironment. **(A)** A number of bacteria are significantly associated with non-chronic wounds using indicator species analysis. Indicator values of non-chronic (blue lines) and chronic (red lines) wounds are shown for the following bacteria over time as non-chronic wounds heal: **(B)** *Cutibacterium acnes*, **(C)** *Achromobacter* sp., **(D)** *Delftia* sp., **(E)** *Streptococcus* sp., **(F)** *Escherichia coli* and **(G)** *Staphylococcus epidermidis*. Non-chronic wound, *n* = 40; chronic wound, *n* = 37. *p < 0.05, **p < 0.01, ***p < 0.001.

Indicator species analysis was used to determine which bacterial species in the wound model were strongly associated with non-chronic and chronic wounds. Indicator values were calculated as (*fidelity* X *specificity*), where the fidelity of a species was its relative abundance and specificity of a species was the relative frequency or the number of samples within the group where the species was detected (Figures 7A, 8). Thus, this analysis considered both the strength and generality of colonization by microbes across different environments. Several bacterial species were found to be consistently associated with chronic wounds compared to non-chronic wounds (Figure 7). Wound tissues that were infected with aggressive biofilm-forming pathogens, such *P. aeruginosa* (Figure 7B), underwent significant damage especially when strong biofilm was present. *P. aeruginosa* became a strongly indicator of chronic wounds after D2 and by D20, chronic wounds are fully developed. *P. aeruginosa* was able to also colonize non-chronic wounds, but the indicator values in these healing wounds were significantly lower in comparison to the values representing the species colonizing biofilms in chronic wounds. Similarly, *E. cloacae* could also colonize both non-chronic and chronic wounds until it was the predominant bacterial species in the wound community in some mice (Figure 7C) but was more strongly associated with chronic wounds. *C. frankenforstense* colonization in the wound began shortly after injury and indicator values in the wound increased until it peaked at D15. Unlike *P. aeruginosa* and *E. cloacae*, this bacterial species did not frequently colonize non-chronic wounds (Figure 7D). *Acinetobacter* sp. (Figure 7E) had a similar indicator values profile as *P. aeruginosa* (Figure 7B). The bacterial trend in chronic wounds increased exponentially after D2 and remained high once biofilm began to form, protecting and supporting the bacteria so it could continue to infect the wound. In non-chronic wounds, the indicator values were very low, suggesting that this species did not colonize the wound well, perhaps due to competition with other species in the wound as the wound heals. *S. xylosus* was a biofilm-former that could quickly infect a chronic wound once an injury was made as specified by high indicator values at D2 and D3 (Figure 7F). However, as seen in individual bacterial mouse profiles (Figures 4D, 6E), stronger biofilm-forming bacteria such as *E. cloacae* and *P. aeruginosa* could out-compete *S. xylosus* until it virtually disappeared from the chronic wounds. Indeed, the indicator value began to decrease at D3, at the same time when *P. aeruginosa, E. cloacae* and *Acinetobacter* sp. began to dominate the wound. After D5, the indicator values between chronic wounds and non-chronic wounds were not significantly different. In non-chronic wounds, *S. xylosus* slowly increased, but much like *C. frankenforstense* and *Acinetobacter* sp., it did not strongly colonize the wound. The indicator value trends of *B. paralicheniformis* was higher shortly after injury but was unable to exist in a community with other strong biofilm-formers (Figure 7G) much like *S. xylosus* (Figure 7F).

### Bacteria That Colonize Non-chronic Wounds Can Be Used as Indicators of Healing

A number of bacterial species were found to be significantly associated with resolving wounds (Figure 8). One of these bacterial species *C. acnes*, formally known as *Propionibacterium acnes*, is an abundant commensal Gram-positive anaerobic bacteria found on human skin (Achermann et al., 2014), especially in the sebaceous glands. *C. acnes* has been associated with skin conditions such as acne and an opportunistic pathogen in post-operative infections in humans (Achermann et al., 2014). However, this species was not commonly associated with human chronic wounds. The indicator value for *C. acnes* in the days following injury increased only in non-chronic wounds while in chronic wounds the value remained very low, showing that *C. acnes* was not significantly associated with chronic wounds in this model system (Figure 8B). While the indicator value trend increased in non-chronic wounds, the indicator value of *C. acnes* was also dynamic over the course of wound healing. *Achromobacter* sp. consisted of non-fermenting Gram-negative bacteria. The indicator value for *Achromobacter* sp. increased significantly starting from D2 in non-chronic wounds compared to chronic wounds (Figure 8C). The high indicator value was sustained through wound healing. Similar to *C. acnes*, the indicator value for *Achromobacter* sp. was very low in chronic wounds, suggesting it cannot survive in a population with other biofilm-forming bacteria. *Delftia* sp., Gram-negative aerobes, had indicator values consistently high healing wounds compared to chronic wounds (Figure 8D). Although *Delftia* sp. could be found in human chronic wounds, they are not common (Wolcott et al., 2016). *Streptococcus* sp. were Gram-positive species and could be commonly found in human chronic wounds (Table 1). The *Streptococcus* sp. in the wound model was significantly associated with non-chronic wounds (Figure 8E). While the indicator values between non-chronic and chronic wounds were similar between D0 and D2, the values were higher in non-chronic wounds compared to chronic wounds from D10. *Streptococcus* sp. was not significantly associated with chronic wounds in the same manner as the other species previously shown above. *E. coli*, a Gram-negative facultative anaerobe, was significantly associated with non-chronic wounds (Figure 8F). Indicator value analysis showed that *E. coli* had significantly higher indicator values throughout the 20 days. Conversely, indicator values for chronic wounds significantly decreased after 72 hrs. *S. epidermis* was a Gram-positive bacterium that was found prevalently in human skin microflora. In the wound model, *S. epidermis* was significantly associated with non-chronic wounds, especially in the early days between D1 and D5 (Figure 8G). After D5, this species began to disappear until D15 at which time it began to return in both non-chronic and chronic wounds. In chronic wounds, indicator values were low, suggesting that this bacterial species was not highly associated with the microenvironment of chronic wounds.

### Comparison of Indicator Values for Bacteria Present in Chronic and Non-chronic Wounds

To identify bacterial species that are associated with a microenvironment that lead to either chronic wound development or wound healing, the dataset was divided into “early” timepoints between D0 and D5 and “late” timepoints between D5 and D20 (Table 2). Indicator value analysis showed that several bacteria, *S. xylosus, Bacillus* sp., and *E. faecalis*, were significantly associated with the early wound microenvironment that resulted in chronic wound development. Conversely, *P. aeruginosa, C. frankenforstense, E. cloacae*, and *Acinetobacter* sp. were identified as late indicators. These bacteria significantly associated with the microenvironment of fully developed chronic wounds that have strong biofilm formation. Several species were identified to be highly associated with the wound microenvironment of non-chronic wounds, including *Delftia* sp., *L. murinus, Turicibacter* sp., and *S. epidermis*. These bacteria species were identified to be significant later during the healing process.

**Table 2:** Bacterial bioindicators for non-chronic and chronic wounds.

**Early indicators of chronic wounds**	***p*-value**	**Late indicators of chronic wounds**	***p*-value**
*Staphylococcus xylosus*	0.001	*Pseudomonas aeruginosa*	0.001
*Bacillus* sp.	0.001	*Corynebacterium frankenforstense*	0.033
*Enterococcus faecalis*	0.048	*Enterobacter cloacae*	0.001
*Staphylococcus nepalensis*	0.001	*Acinetobacter* sp.	0.036
*Erysipelothrix rhusiopathiae*	0.05		
*Lachnospiraceae*	0.045		
*Mucinivorans hirudinis*	0.045		
**Early indicators of non-chronic wounds**	***p*****-value**	**Late indicators of non-chronic wounds**	***p-*****value**
*Delftia* sp.	0.001	*Cutibacterium acnes*	0.001
*Lactobacillus murinus*	0.015	*Streptococcus* sp.	0.001
*Turicibacter* sp.	0.004	*Achromobacter* sp.	0.001
*Staphylococcus epidermidis*	0.026	*Escherichia coli*	0.014
*Weissella paramesenteroides*	0.001	*Lawsonella clevelandensis*	0.007
*Paenibacillus* sp.	0.04	*Lactobacillus crispatus*	0.049
*Bacteroidales*	0.004	*Pseudomonas* sp.	0.027
*Lactobacillus* sp.	0.005	*Corynebacterium choanis*	0.012
*Massilia oculi*	0.046		
*Massilia* sp.	0.003		
*Planococcus* sp.	0.029		

## Discussion

Sequencing the bacterial ITS rRNA gene over the course of healing or chronic wound development, revealed that the wound microbiome was complex, dynamic, and could be altered or disturbed by high levels of OS. When wounds had normal levels of OS as part of the wound healing program, the wound microbiome was diverse and dynamic with many bacterial species present. Although pathogenic bacteria species could be found in the healing wounds, these species did not form biofilm in the wound. When inhibitors for antioxidant enzymes were administered, resulting in excessive levels of OS induced beyond the scope of the normal wound healing process (Kim et al., 2019), detrimental changes to the wound microenvironment supported the colonization of opportunistic biofilm-forming bacteria such as *P. aeruginosa, E. cloacae*, and *S. xylosus*. As a consequence, while several bacteria species were present in the wound the first few days after injury, competition between commensal bacterial species unable to withstand these conditions and the strong biofilm-forming opportunistic colonizers resulted in a decrease in overall diversity of the wound microbiome over time with biofilm development. Individual wound profiles of healing and chronic wounds demonstrated that mice with identical genetic backgrounds could have distinct and personalized bacterial colonization depending on the levels of OS in the wound. Although the same species dominated chronic wounds, no two wound profiles were the same. Indicator value analysis showed that several bacteria found in the model were highly associated with either healing wounds or wounds that developed into chronic wounds. These bacteria followed different time trends in healing wounds and chronic wounds, illustrating the complexity of bacterial interactions that resulted in these distinct patterns. Bacteria highly associated with non-chronic wounds may play an important role in wound healing, by stimulating the immune response or initiating wound healing processes. Bacteria that have been identified as bioindicators of healing wounds may also play a role in interacting with opportunistic pathogens, to control their growth and prevent them from strongly colonizing the wound. A number of bacterial species sequenced in the model were also found in human chronic wounds (Table 1), demonstrating the model's ability to recapitulate conditions for chronic wound development with the formation of biofilm from clinically relevant bacteria that was naturally found in the skin microflora.

Previously culture-based methods have been used to identify important wound pathogens. However, culture-dependent techniques were limited in their ability to evaluate complex microbiomes due to the variety of specific culture conditions required to grow different bacterial taxa (Temmerman et al., 2004). Culture-independent metagenomic techniques has been used to describe the microbial diversity of complex communities, such as the wound-associated microbiome (Ye et al., 2008; Hodkinson and Grice, 2015). One of the most common methods is high-throughput sequencing of bacterial 16S ribosomal RNA (16S rRNA) genes (Hannigan et al., 2014; Misic et al., 2014; Hodkinson and Grice, 2015). In this study, the genomic ITS rRNA gene was targeted over traditional 16S rRNA sequencing in order to obtain taxonomical information about the bacterial community in the chronic wound model. The ITS region offered species and subspecies-level resolution because this region could harbor higher sequence variations (Ruegger et al., 2014). While sequencing the 16S rRNA revealed the complicated nature of microbiomes, the data was commonly represented at the genus taxonomical level which made interpretation of the data difficult because bacteria acted as either pathogens or commensal bacteria at the species level. Other bacteria needed to be identified at the subspecies or strain levels to identify its status of pathogenicity (Kalan et al., 2019).

Wound profiles of individual mice that showed strong biofilm-forming bacteria colonization, from species such as *P. aeruginosa* and *E. cloacae*, were capable of dominating the wound microbiome in non-chronic wounds and chronic wounds alike. These two species are very important clinical bacteria found in chronic wounds of humans (Dowd et al., 2008a; Serra et al., 2015). *P. aeruginosa* especially is a notorious biofilm-forming bacterium in human chronic wounds and is important in other diseases such as cystic fibrosis (Winstanley et al., 2016). This is of concern because strong colonization of the wound should be indicative of infection and biofilm formation. However, analysis of bacterial trends using indicator value analysis showed one significant difference between bacteria prominently found in non-chronic wounds as opposed to chronic wounds: bacteria preferentially colonizing chronic wounds occupied the bacterial community at high abundances when compared to non-chronic wounds. In chronic wounds, biofilm created by the opportunistic pathogenic bacteria significantly enhanced the ability of the bacteria to colonize the wound. In the biofilm, nutrients and oxygen were effectively available for the bacteria harbored inside, allowing populations of many bacterial species to significantly increase as observed with *P. aeruginosa, E. cloacae, C. frankenforstense* and *S. xylosus*.

Analysis of the bacterial microbiome data revealed a subset of mice with low populations of the strong biofilm-formers such as *P. aeruginosa* and *Acinetobacter* sp. When *P. aeruginosa* was present in low percentage of the bacterial population, *E. cloacae* was able to dominate the wound microbiome. *E. cloacae* is a nosocomial opportunistic pathogen found in the environment as well as on human skin and is frequently identified in the biofilm of the wounds with some strains already harboring anti-microbial resistance genes (Davin-Regli and Pagès, 2015). In our model system, *E. cloacae* was present in high proportions in both non-chronic and chronic wounds. In wounds that did not have high levels of OS, *E. cloacae* did not form biofilm and we did not observe delays in healing. However, when the wounds are administered inhibitors for catalase and glutathione peroxidase to induce high levels of OS and become chronic, the wounds formed strong biofilm (Dhall et al., 2014; Kim et al., 2019). These studies suggest that high levels of OS are necessary to activate or allow for the formation of biofilms by opportunistic pathogens and that the ability to adapt to these high OS conditions may determine which opportunistic pathogen is able to effectively colonize and develop biofilms in the wound.

In our *db/db*^−/−^ model, we were able to identify key pathogenic bacteria such as *P. aeruginosa, E. cloacae, C. frankenforstense, Acinetobacter* sp., and *S. xylosus* that are present in the biofilm of the chronic wounds. These bacteria are clinically relevant because they are found in human chronic wounds and create biofilm which complicates and significantly delays healing (Table 1). The infection and production of biofilm in our model is derived only from the manipulation of the redox state of the wound tissue in diabetic, obese mice, with the participating bacteria originating naturally from the skin microbiota (Dhall et al., 2014; Kim et al., 2019). The infection and biofilm production can be observed from the initial stages in the chronic wound model so that mechanisms of pathogenicity in these bacteria can be understood *in vivo*. This is a significant advantage since chronic wounds in humans are observed by physicians and wound care specialists weeks, sometimes months or even years, after the initial injury.

Several bacterial species that are used as probiotics to treat disease in humans are also present in the bacterial microbiome in our mouse model (Table 3). Microbiome studies in the gut have led to the development of probiotics, a cocktail of bacteria that can reverse disease progression by replacing harmful bacteria with beneficial ones. It has been shown that bacterial interactions between *Lactobacillus plantarum* with *P. aeruginosa* in a burn mouse model lead to inhibition of *P. aeruginosa* colonization, resulting in better wound healing outcomes such as tissue repair and enhanced phagocytosis of *P. aeruginosa* (Valdéz et al., 2005). Consuming probiotics supplements consisting of several *Lactobacillus* sp. were able to improve clinical outcomes in patients with diabetic foot ulcers (Mohseni et al., 2018). Ethyl acetate extract of an *Achromobacter* sp. from entomopathogenic nematodes were found to have significantly antibacterial properties against biofilm-forming bacteria such as *P. aeruginosa* and *S. aureus*. Specific cyclic dipeptides, in combination with ampicillin, inhibited biofilm formation while also increasing production of anti-inflammatory cytokines from peripheral blood mononuclear cells (Deepa et al., 2015). Even though species such as *Achromobacter xylosoxidans* are implicated in nosocomial infections, infection with this species in skin is thought to be very uncommon (Tena et al., 2014). However, this species was found to be infecting burn wounds and is an emerging pathogen of cystic fibrosis (Parkins and Floto, 2015; Schulz et al., 2016).

**Table 3:** Bacteria used as probiotics in humans.

**Bacteria**	**References**
*Enterococcus faecalis*	Gao et al., 2015; George Kerry et al., 2018
*Lactobacillus* sp.	Scales and Huffnagle, 2013; Sáez-Lara et al., 2016; Lukic et al., 2017; Mohseni et al., 2018
*Lactobacillus johnsonii*	Markowiak and Slizewska, 2017
*Lactobacillus reuteri*	Lukic et al., 2017; Markowiak and Slizewska, 2017; George Kerry et al., 2018
*Streptococcus* sp.	Sáez-Lara et al., 2016

Whereas research in pathogenic bacteria have resulted in the identification of mechanisms in which bacteria may interfere with wound healing (Schmidtchen et al., 2003), mechanisms to understand the beneficial role of probiotics in wound healing are still in their infancy. Bacteria identified to be highly associated with non-chronic wounds (Table 2) may be beneficial by assisting in stimulating the immune system and wound healing processes. A combination of multiple bacterial species with different metabolisms may collectively strengthen the affect the bacteria has on wound healing, since interactions between commensal bacteria may lead to a more diverse set of active metabolites that can stimulate the immune response or wound healing (Li et al., 2018). Bacteria identified as early bioindicators may perform as putative probiotics since they are present in the wound immediately after injury and can interact and compete with opportunistic pathogenic bacteria to prevent them from colonizing the wound and forming biofilm. Bacteria identified as early bioindicators of chronic wounds (Table 2) may have a significant role in the presence of high levels of OS in establishing a wound microenvironment that allows other biofilm forming pathogens to infect and colonize the wounds. These bacteria are of particular interest because late bioindicators of chronic wounds are significant bacterial species isolated and identified in human chronic wounds. To the best of our knowledge, the microbiome of a wound immediately after injury that will develop into chronic wounds has not been surveyed. Analysis on wound microbiomes of human chronic wounds (Table 1) are currently done on mature chronic wounds, and thus, are more similar with bacterial species identified as late chronic wound bioindicators. Comparing the bacterial bioindicators of non-chronic wounds and acute/healing human wounds may assist in focusing research to identify bacterial species that function as putative probiotics, or identify metabolites, that when applied on wounds can stimulate proper wound healing. Conversely, analysis of different types of human chronic wound microbiomes in comparison to the bioindicators of chronic wounds found in our model can focus efforts to understand how early chronic wound bioindicators interact with late chronic wound bioindicators to lead to chronic wound development. In that endeavor, our chronic wound model can be used to understand host–microbe interactions to identify novel mechanisms, pathways, chemicals, or metabolites that can effectively prevent or reverse chronic wound formation.

The microenvironment of human chronic wounds is frequently manipulated in order to eliminate harmful bacteria and facilitate wound healing (Wolcott et al., 2009; Powers et al., 2016). Wounds are often debrided to remove biofilm and dead tissue, and subsequently treated with wounds dressings to protect the wound and stimulate wound healing. Antibiotics are also administered in order to prevent further colonization of biofilm-forming bacteria (Howell-Jones et al., 2005; Lipsky and Hoey, 2009). Despite great efforts put into healing chronic wounds, treatment efficacy has been low with many wounds persisting despite antibiotic therapy, wound debridement, and applications of wound dressings. This is because the underlying cause of chronic wound development is poorly understood, and the treatments applied and therapies used are not targeting the underlying cause. Human wound microenvironments are very complex and many cells that participate in wound healing are dysfunctional in human chronic wounds. Just as important as the wound parameters in patients are the bacteria that colonize the wound. Chronic wounds are rarely colonized by a single pathogenic species. Instead, several “cornerstone” species are observed, including common nosocomial species such as *P. aeruginosa, Enterobacter* sp., *Staphylococcus aureus* and Acinetobacter baumannii. Many species found in chronic wounds are also found in normal skin microflora. While these species may be detected because they are skin components, they may have a role in either promoting wound healing or contributing toward the development of non-healing wounds. Applying broad-spectrum antibiotics, while used to control the population of pathogenic species, also removes from the wound bed species that may positively contribute to healthy healing through competition of pathogenic species. Also important, and underscored in this study, is the longitudinal nature of chronic wound development. Most microbiome studies in human chronic wounds are samples of the wound at only one time. In addition, the timing of sampling is crucial in the days following debridement, a common technique used to clear away necrotic and infected tissue to re-expose healthy tissue. Future microbiota studies in human chronic wounds would benefit from sampling several timepoints.

*In conclusion*, our studies demonstrate distinctive bacterial trends in the microbiome of healing non-chronic and chronic wounds in our diabetic mouse model. In non-chronic wounds, higher levels of bacterial diversity and dynamic colonization were observed over time as the wounds healed. Opportunistic pathogens, capable of forming biofilm, were present in the wound microbiota and did not make biofilm without increased levels of OS. In contrast, chronic wounds had low levels of bacterial diversity and were colonized by strong opportunistic pathogens that formed biofilm and out-competed other bacteria present in the wound microenvironment. Chronic wounds are colonized by *P. aeruginosa* when it is present in the microenvironment. Conversely, *E. cloacae* is the dominating wound bacterial species when *P. aeruginosa* is not present. Indicator value analysis showed that specific bacterial species are highly associated with non-chronic wounds compared to chronic wounds; these bacteria may potentially benefit or facilitate wound healing and the exclusion of pathogenic bacteria. The microbiome studies in this paper show that our chronic wound model provides the correct microenvironment that will enable the study of bacterial interactions that lead to either wound healing or chronic wound development, potentially leading to new methods of controlling biofilm in chronic wounds in order to support wound healing.

## Data Availability Statement

The raw data supporting the conclusions of this article has deposited in the National Center for Biotechnology Information (NCBI)'s Sequence Read Archive (SRA) under the BioProject Accession Number PRJNA623025.

## Ethics Statement

The animal study was reviewed and approved by University of California, Riverside Institutional Animal Care and Use Committee (IACUC).

## Author Contributions

JK and MM-G designed the study and wrote the first draft of the manuscript. JK collected and processed the samples with the assistance of EL. PR generated the OTU table. SV, DJ, and AH assisted in the discussion for the analysis of the data. AH and JB reviewed the manuscript and MM-G worked with JK in the rewriting of the manuscript to its final form.

## Conflict of Interest

The authors declare that the research was conducted in the absence of any commercial or financial relationships that could be construed as a potential conflict of interest.
